# The biomechanical behavior of 3D printed human femoral bones based on generic and patient-specific geometries

**DOI:** 10.1186/s41205-022-00162-8

**Published:** 2022-11-23

**Authors:** Katharina Nägl, Andreas Reisinger, Dieter H. Pahr

**Affiliations:** 1 Division Biomechanics, Karl Landsteiner University of Health Science, Dr.-Karl-Dorrek-Straße 30, 3500 Krems an der Donau, Austria; 2 Institute for Lightweight Design and Structural Biomechanics, TU Wien, Getreidemarkt 9, 1060 Wien, Austria

**Keywords:** 3D printing, Fused-deposition modeling (FDM), Biomechanical testing, Femur

## Abstract

**Background:**

Bone is a highly complex composite material which makes it hard to find appropriate artificial surrogates for patient-specific biomechanical testing. Despite various options of commercially available bones with generic geometries, these are either biomechanically not very realistic or rather expensive.

**Methods:**

In this work, additive manufacturing was used for the fabrication of artificial femoral bones. These were based on CT images of four different commercially available femoral bone surrogates and three human bones with varying bone density. The models were 3D printed using a low-budget fused deposition modeling (FDM) 3D printer and PLA filament. The infill density was mechanically calibrated and varying cortical thickness was used. Compression tests of proximal femora simulating stance were performed and the biomechanical behavior concerning ultimate force, spring stiffness, and fracture pattern were evaluated as well as compared to the results of commercial and cadaveric bones.

**Results:**

Regarding the ultimate forces and spring stiffness, the 3D printed analogs showed mechanical behavior closer to their real counterparts than the commercially available polyurethan-based surrogates. Furthermore, the increase in ultimate force with increasing bone density observed in human femoral bones could be reproduced well. Also, the fracture patterns observed match well with fracture patterns observed in human hip injuries.

**Conclusion:**

Consequently, the methods presented here show to be a promising alternative for artificial generic surrogates concerning femoral strength testing. The manufacturing is straightforward, cheap, and patient-specific geometries are possible.

## Background

The field of additive manufacturing (AM) is advancing rapidly and has gained significant importance in recent years. Meanwhile, AM is particularly dominant in the fabrication of prototypes, as well as in small-scale production, and is used in a wide range of applicational fields, such as aerospace and automotive industries [1]. It even found its way into the food industry [2] and medical applications, as it proves to be of use in orthopedics [3–7].

There are various AM manufacturing processes like Material Extrusion based Fused Deposition Modeling (FDM), Vat Photopolymerization based Stereolithography apparatus (SLA), Powder Bed Fusion based Selective Laser Sintering (SLS), Binder Jetting (BJ), and Material Jetting (MJ) to only name a few [8]. The simplest, cheapest, and most widely available method is FDM [9] but various techniques are used for the fabrication of prototypes.

Besides orthopedic applications, AM comes in handy in the fabrication of anatomical models [10–14]. For example, researchers 3D printed breast phantoms via MJ based on mammographic projections [13], and also fabricated a lower limb including femoral vessels using BJ for teaching and training purposes [14]. 3D printed models not only can be used in teaching anatomy but also in the evaluation of surgical procedures [15–17]. Therefore, 3D printing was also used for a whole torso model including organs simulating the pneumoperitoneum, where laparoscopic manipulations could be performed successfully [18]. Meanwhile, MJ is used for surgical guidance before and during complicated surgeries such as the resections of small invisible tumor metastases [19] or during cardiac surgery for intraoperative orientation [20], as well as a navigational template for complicated tibia plateau fracture surgery [21].

Besides the printing of surgical guidance tools, AM techniques such as FDM, SLS, SLA, and BJ were used independently by researchers to manufacture 3D printed models of different bone types and tested the accuracy of the models in comparison to their imaging [10, 11]. They showed that the method could reproduce the topographical features of the bones correctly. Additional to the printing of the clavicle and metatarsal [10], also other bones such as the occipital bone, the left temporal bone, and sphenoid bone were successfully printed with good detail [12]. However, only the surface of the bones was modeled accurately without taking the architecture of trabecular bone into account. Additionally, Cherkasskiy et al. started the process of more thoroughly modeling the trabecular bone by taking into account the infill density that represented the spongiosa [19] in surgical planning models printed using FDM [22].

However, none of the studies discussed above did investigate the biomechanical behavior of the 3D printed parts. Thus, these models were not usable for biomechanical studies where mechanical similarity like stiffness or strength plays a role. Therefore, the necessity of real human bones for testing new implant systems, screws, and other medical applications, is still present. But since donor bones are rare and often accompanied by ethical concerns, the demand for artificial bones has increased. Another concern is the variability of cadaveric bones, not only due to geometrical aspects but also due to age and health differences. Another type of artificial bone is made of thermoplastics, polyurethane foam (PU) or fiber-reinforced epoxy resin fabricated through molds. A well-known example of such artificial bones with generic geometry is the Sawbone (Sawbone®, Vashon Island, USA). But these artificial bones are of standardized, generic geometry with mechanical characteristics mimicking natural bone to some extent [23, 24]. These characteristics have a positive effect on the reproducibility of mechanical experiments. Artificial bones already are used to test and improve implant systems, such as intramedullary nailing systems [25, 26], locking plate systems [27], bone screw purchase [28, 29], or fracture fixation constructs [30]. However, before the testing of implants a critical evaluation of the available synthetic bones is necessary as significant differences have been observed in their biomechanical behavior [31, 32]. In addition, the different materials used for artificial bones, such as polyurethane foam or a composite material consisting of short fiber reinforced epoxy resin must be considered, since the latter can result in cost-intensive samples. With the generic geometry of commercially available bone surrogates, no patient-specific experiments or studies are possible. Where the capability of AM to 3D print patient-specific geometries would come in handy. However, to the best of the author’s knowledge, no studies were found in the literature, which deal with the biomechanical behavior of 3D printed bone surrogates, especially femoral bone strength.

Therefore, the present study is looking for a cheap and fast AM method of 3D Printing biomechanical models where stiffness and load-bearing play a role. The work focuses on FDM printed bones and investigates the possibility of using such AM alternatives to producing artificial femoral bone surrogates. They are based on generic geometries taken from commercial bone surrogates as well as patient-specific geometries from real donor bones and the biomechanical performance is compared based on lab experiments of the stance phase.

## Methods

### Materials

**Fig. 1:**
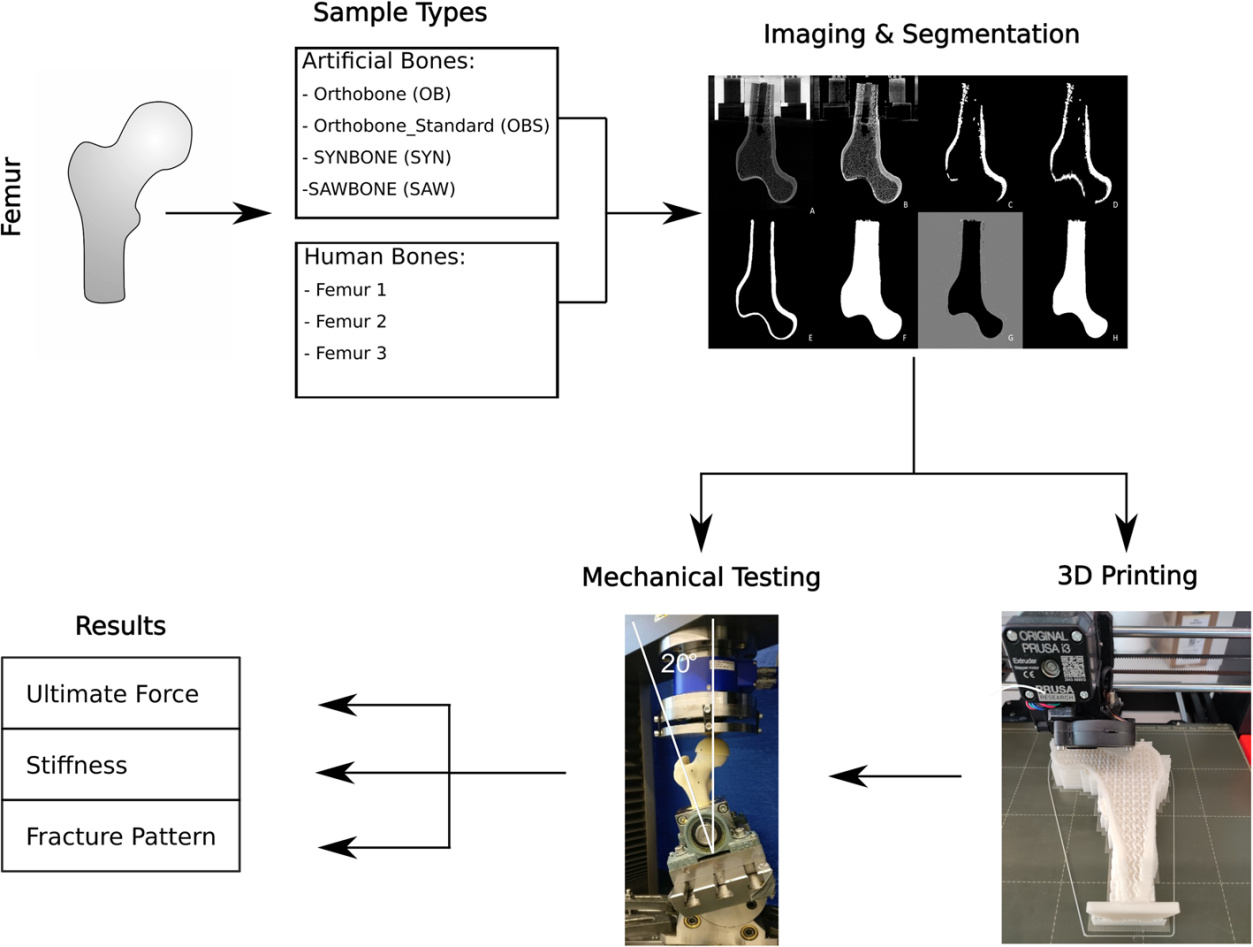
Overview of the workflow. Starting with the different sample types of generic (commercial) and real (human) femoral geometries. Followed by the imaging and segmentation of the μ-CT scans, preparing them for 3D printing and mechanical testing to obtain the ultimate forces, stiffnesses, and fracture pattern of the sample groups

In this study, two groups of samples of femurs were investigated. One group is comprised of generic geometries based on four different commercially available artificial bones. The second group used real femoral geometries based on Quantitative Computed Tomography (q-CT) data sets taken from a previous study done by Dall’Ara *et. al*. [33]. Four different commercially available human femoral surrogate bones were prepared for micro-computed tomography (μ-CT), followed by image segmentation, 3D printing, and biomechanical testing. An overview of the production process is presented in Fig. 1.

The artificial bones Orthobone_Standard (ORTHObones Standard Oberschenkelknochen, rechts Artikel-Nr.: 1019601) and Orthobone (ORTHObones Premium Oberschenkelknochen (Femur), rechts Artikel-Nr.: 1005117 [W19121]) sharing the same geometry but varying in foam density were purchased from 3B Scientific GmbH (Ludwig-Erhard-Str. 20, 20459 Hamburg, Germany) and were further abbreviated as OBS and OB. The third generic bone, SYNBONE®, SYN (right femur with distal canal opening 2230) was purchased from SYNBONE AG (Tardisstrasse 199, 7205 Zizers, Switzerland) and varies in geometry but has a similar foam density to the OB bone inside. These three types of generic geometry bones are all based on PU foam. A composite bone (Femur, 4th Gen., Composite, 17 PCF Solid Foam Cancellous, Medium (SKU: 3403)) was chosen as a fourth generic bone, which was purchased from Sawbone® (Sawbone®, Vashon Island, USA) and will be further abbreviated as SAW.

**Fig. 2:**
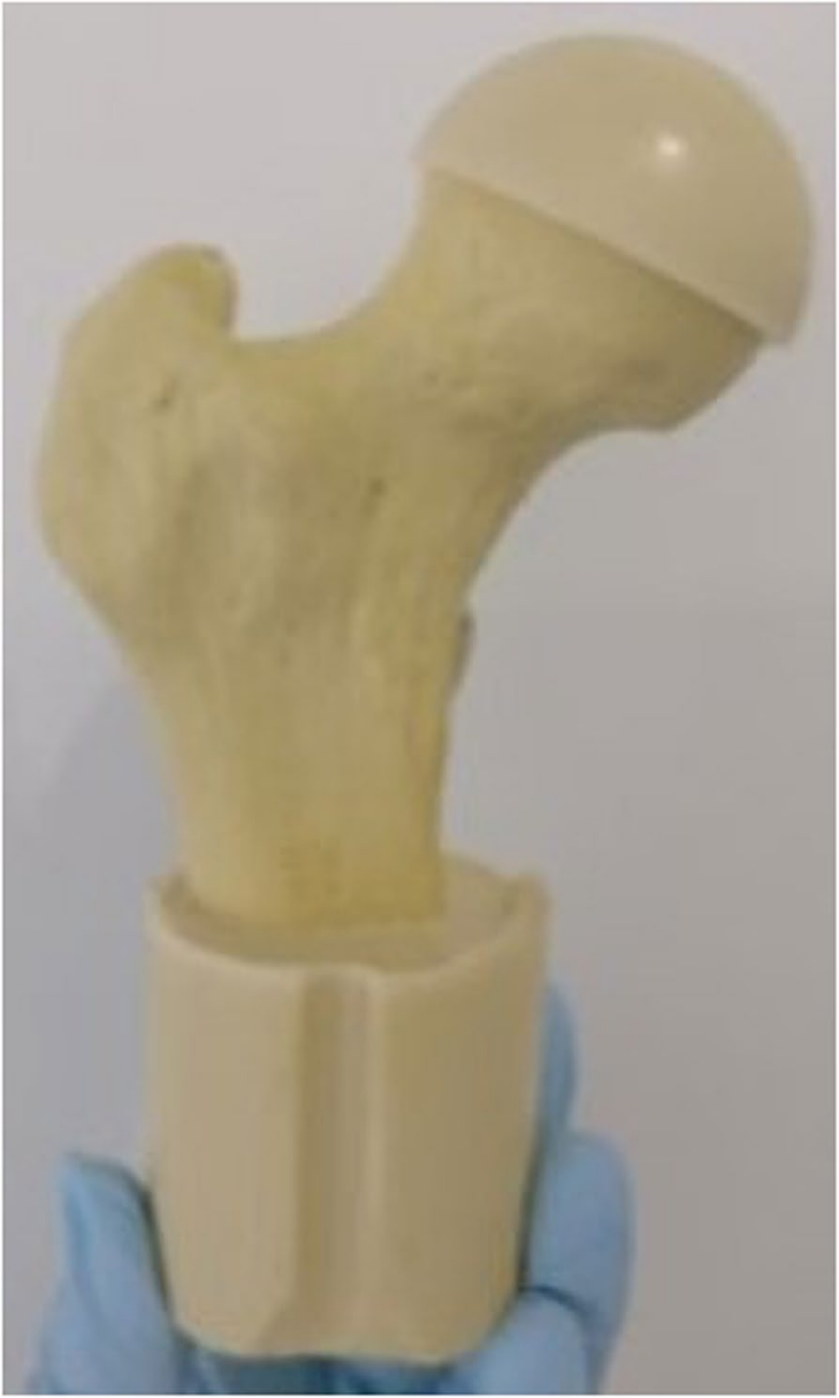
Embedded truncated generic Femur in preparation for μ-CT scanning

Each commercial femur was aligned in the mechanical axis and truncated 15 cm from the caput femoris down to the corpus femoris [34]. The bottom and the femoral head of the cut-off femur were embedded in polyurethane (SG 141/4 and PUR 145 hardener from FDW Handels ges.m.b.H., 8940 Liezen, Austria) in preparation for μ-CT scanning and mechanical testing as shown in Fig. 2.

### Imaging and segmentation

The generic bones were scanned using a Bruker Skyscan 1173® (Bruker Corporation, Billerica, MA 01821). Scanning parameters were chosen as a voltage of 65 kV, with a probe current of 75 mA and the resolution was set to 70 μm. The obtained scans were reconstructed using NRecon® software (Micro Photonics Inc., Allentown, PA 18104). The obtained μ-CT images were saved as DICOM files for further image processing.

**Fig. 3:**
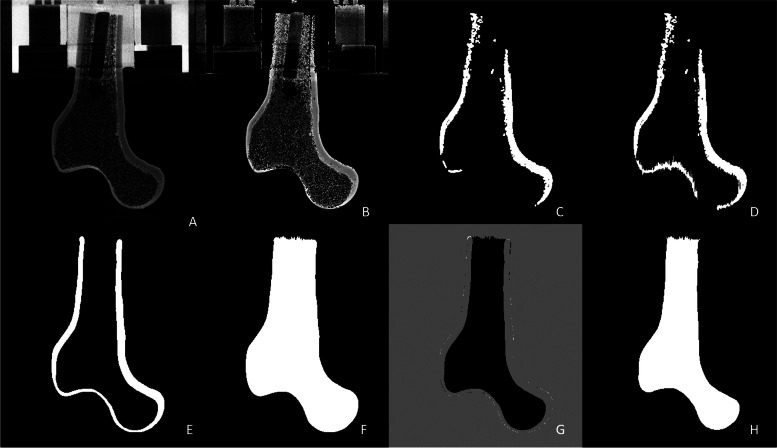
Steps of Segmentation in terms of Midplanes of SYN. **A** rescaled image showing the truncated bone with its embedding and the sample holder used for the μ-CT scanning **B** usage of a global threshold to get rid of the embedding as well as the sample holder **C** usage of a morphological filter to separate the cortex and the spongiosa **D** first manual segmentation in 3D Slicer **E** smoothed final manual segmentation of the cortex **F** whole bone mask **G** subtracted cortex from the whole bone to get the spongiosa **H** grey value inverted spongiosa

To be able to print the scanned bones, it is necessary to transform the DICOM files into triangulated surfaces (STL files). To achieve an accurate representation of the scanned object, two STL files were necessary for each bone, namely the periosteal and endosteal surface. The STL files are obtained by segmentation into a trabecular and cortical volume, which were performed using a combination of medtool 4.5 software (Dr. Pahr Ingenieurs e.U., Pfaffstätten, Austria) and 3D Slicer v4.11.20200930® [35]. By resizing the images first, faster processing was ensured. Furthermore, midplane images of the bone were created to visualize the femur and its embedding as shown in Fig. 3A based on the SYN sample. Secondly, a global threshold was used to segment the bone structure from its surrounded embedding and the holder for the μ-CT scans (Fig. 3B). In a second step, the remainder were removed by using a morphological operation to segment the cortex (Fig. 3C). Due to a thinner cortex in the *Fossa trochanteric* region, manual segmentation (Fig. 3D) was necessary. The cortex was automatically smoothed to achieve clear borderlines as shown in Fig. 3E and then exported as an STL file. To obtain a separate 3D model of the spongiosa the cortex had to be subtracted from the whole bone structure (Fig. 3F) and the grey values had to be inverted (Fig. 3G). The result of the segmentation for the spongiosa is shown in Fig. 3H and was exported as an STL file as well.

### 3D printing

Since in this study the generic geometry bones and the real human ones should be modeled in terms of mechanical similar behavior, two major questions emerged after exporting the STL files.

On the one hand, the stiffness of the foam representing the artificial spongiosa had to be determined to match a mechanically proper infill density for the 3D printed bones. On the other, the real human bone spongiosa and the artificial spongiosa had to be matched to an infill density of the 3D printed analogs to achieve a similar mechanical representation.

### Infill density estimation

The estimation of the stiffness of the foam of the artificial bones was based on 10 mm edge length cubes, which were milled out of the distal end of the femur. Mechanical testing was performed on a Z030 machine (ZwickRoell GmbH & Co. KG, D-89079 Ulm, Germany) with a compression rate of 1 mm/min [36] until failure. This compression rate was chosen according to the ISO 844 [36] and considered to be quasi static and slow enough to have a vanishing viscous influence. The corresponding Young’s modulus were calculated based on the recorded force-displacement curves and are listed in Table 1 for OBS, OB, and SYN samples. Concerning the SAW sample, the manufacturer provided the mechanical information of interest, therefore no test was performed on the infill of the composite bone.

**Table 1:** Estimated *Young’s modulus* of the commercial bone foam and the corresponding infill densities for the 3D printed analogs

Sample	Young’s modulus in MPa	3D printed Infill density in %
**OBS**	9.06 ± 0.41	7
**SYN**	33.67 ± 7.12	12
**OB**	35.6 ± 12.07	12
**SAW**	155	27

**Fig. 4:**
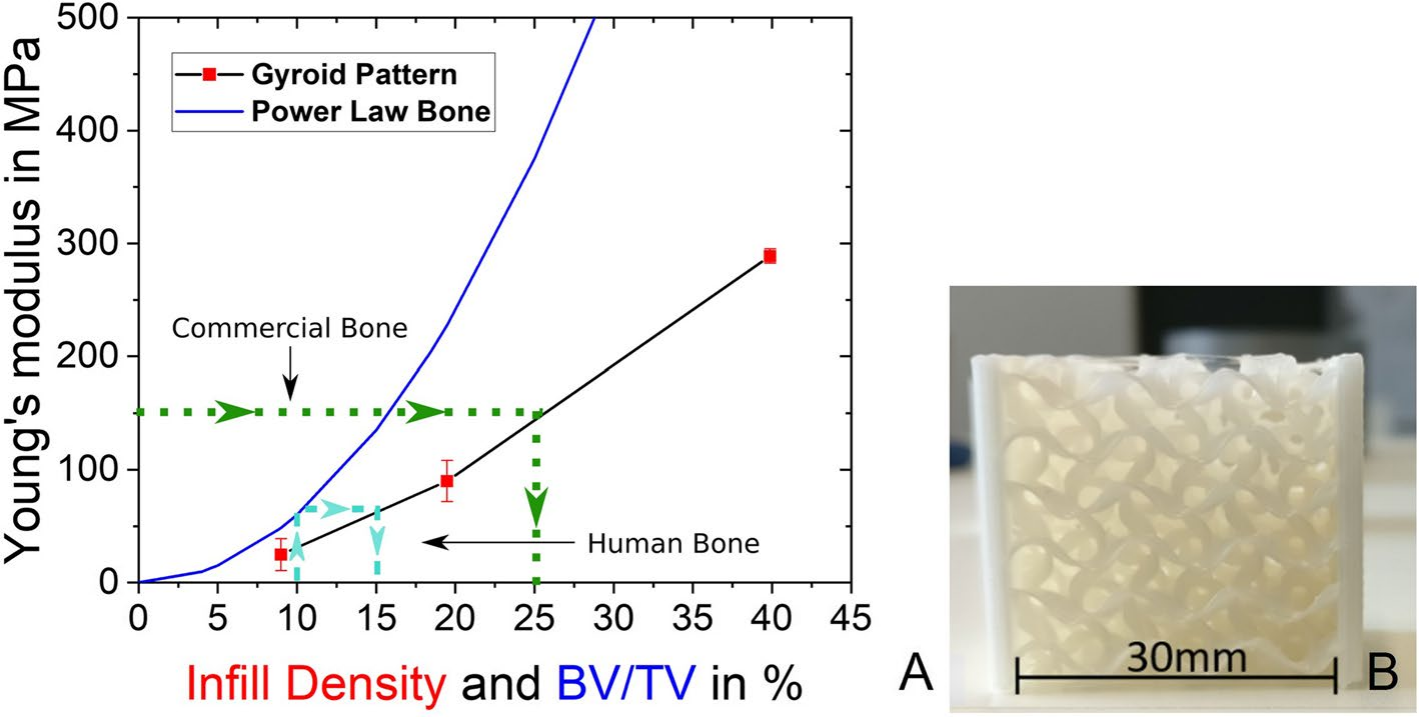
**A** Established Power Law connecting the *Young’s modulus* of bone with the volume fraction of gyroid pattern infill density and BV/TV and experimental curves from cubical PLA samples with varying Gyroid infill. Furthermore, an example of the estimation process of the infill density of the artificial (green dotted line) and human (turquoise dashed line) bones **B** gyroid pattern infill test cube (tested orthogonally to the printing direction)

For the printing, the gyroid pattern was chosen to mimic spongiosa patterns in femoral bones according to studies found in literature [37, 38]. It was necessary to first test the mechanical performance of different infill densities of the gyroid pattern, to be able to match the results of the foam tests (Table 1) to the proper infill density in mechanical terms. Therefore, three different densities (10, 20, and 40%) of 30x30x30 mm edge length cubes were printed (Fig. 4B) and tested mechanically. Resulting in Young’s modulus in the longitudinal direction of 24.73 ± 14 MPa for 10%, 89.91 ± 18 MPa for 20%, and 288.79 ± 6 MPa for 40% infill density (Fig. 4A red points). This established relationship (Fig. 4A) enables to directly link the Young’s modulus to different infill densities of the gyroid pattern and will be used to match a mechanical representative infill density to the 3D printed bones.

Followed by the establishment of a power law (Eq. 1) based on these three different infill densities, and the findings of Dall’Ara *et. al*. [33]. This should provide a relationship between the Young’s modulus of bone samples, the infill density of the gyroid pattern, and subsequently also to the average $\frac{BV}{TV}$ as shown in the blue curve in Fig. 4A.

1 $${E}_{\textrm{eff}}={E}_{\textrm{bone}}\ast {\rho}^k$$

Where *E*_eff_ is the resulting Young’s modulus in MPa, *ρ* is either the infill density of the tested cubes in case of the artificial bones or the volume fraction of bone volume (BV) over total bone volume (TV) in case of modelling the real bones. Many choices of power law parameters can be found in literature like in Munford *et. al*. [39] where a comparison between different power laws was made. In this study, *E*_bone_ was assumed to be 6000 MPa and *k*, the power-law factor, was chosen to be 2. Looking at the resulting density relationship in comparison to Munford *et. al*. [39], this assumption gives somehow a “mean” fit to this huge variation of data within a density range from 0 to 40%. In other words, slightly different power law parameters would give nearly the same fit in this density range, and, thus, the same outcome as explained in the following.

Based on the established relationship shown in Fig. 4A, the tested foam cubes could be matched to a mechanically representative infill density. This was done by looking up the calculated Young’s modulus of the commercial foams (Table 1) in the graph and first matching it with the bone power law curve. In the next step, this Young’s modulus was looked up on the gyroid pattern cube curve (green dotted line in Fig. 4A) and the corresponding infill density was determined. Due to the established relationship, infill densities for the generic 3D printed analogs were chosen between 7% and 27% as shown in Table 1.

The infill density in 3D printing can be interpreted as a volume fraction filled with material. Wherefore, the same considerations concerning the “infill density” of bone in terms of trabecular bone inside the femoral head of the real bones were made. The real bones were taken from a previous study [33] where the images are already scaled between 0 (no bone, only air) and 1064 (100% bone). In the next step, the sum of all grey values in the trabecular region was calculated (∑*GV*_1064_). Followed by setting all grey values of voxels in the trabecular region to 1 and everywhere outside to 0. This was done to be able to calculate the number of voxels of the trabecular region (∑*GV*_*B*_). Finally, the bone volume fraction follows from:

2 $$\frac{\overline{BV}}{TV}=\frac{\sum {GV}_{1064}}{1064\ast \sum {GV}_B}$$

Where $\frac{\overline{BV}}{TV}$ is the mean volume fraction of the trabecular region.

Based on the calculated values listed in Table 2 and the before-established power law (Fig. 4A) infill densities of the gyroid pattern were matched to represent the real human bones in mechanical terms. Wherefore the calculated $\frac{\overline{BV}}{TV}$ was looked up in the graph of Fig. 4A and first matched to an equivalent Young’s modulus of the bone (turquoise dashed line). Followed by finding the corresponding infill density of the gyroid pattern, which would represent this specific Young’s modulus. This procedure led to gyroid pattern infill densities of 16% and 27% as listed in Table 2.

**Table 2:** Estimated BV/TV average of trabecular region and corresponding infill densities for the 3D printed analogs

Sample	∑*GV*_1064_	∑*GV*_*B*_	$\frac{\overline{BV}}{TV}$ in %	Estimated Young’s modulus in MPa	3D printed Infill Density in %
**Femur 1**	1.74 ∗ 10^8^	4.41 ∗ 10^6^	15.8	~ 150	27
**Femur 2**	7.52 ∗ 10^7^	2.74 ∗ 10^6^	11	~ 60	16
**Femur 3**	9.85 ∗ 10^7^	3.82 ∗ 10^6^	10.3	~ 60	16

### Slicing and printing parameters

**Fig. 5:**
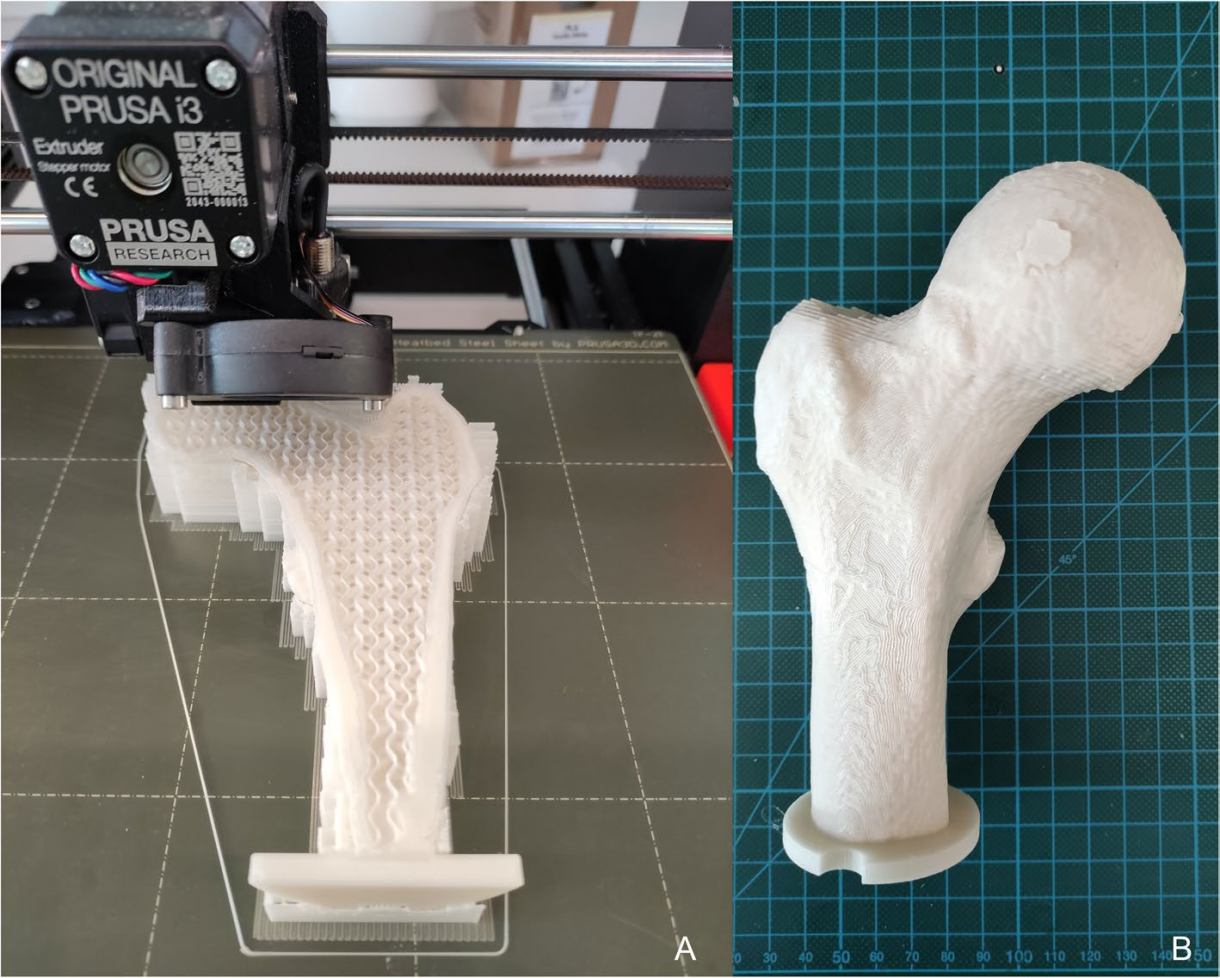
Printing Process and finally 3D printed bone example (Femur1). **A** Example of a 3D printed bone during the printing process with support. The variable cortex thickness with 100% linear infill is shown as well as the gyroid pattern which represents the artificial spongiosa. At the distal end, a plate was added to place the bone before embedding. **B** final 3D printed bone after removal of the supporting structure

Slicing was done in PrusaSlicer 2.1.1® (Prusa Research a.s., Prague, Czech Republic). Therefore, the STL files of the cortex (Fig. 3E) and spongiosa (Fig. 3H) were imported and sliced separately. Regardless of the geometry or the manufacturer, a linear infill pattern with 100% infill density and the same extrusion width (EW) was used for all cortices as listed in Table 3. For the spongiosis, the same layer and perimeter settings were chosen (Table 3), only the infill pattern (gyroid) and infill densities were varied as discussed above. Furthermore, a larger extrusion width (EW) of 0.6 mm (default 0.45 mm) was chosen to improve layer adhesion. Also, the printing temperature was increased to 225 °C to enhance cohesion and prevent layer splitting during compression tests. Besides these settings, 0.20 mm QUALITY MK3 printer settings, original Prusament Filament® (Vanilla White® PLA), and Original Prusa i3 MK3® as a printer were used (Fig. 5).

**Table 3:** Common printing parameters for the cortices and spongiosis with the extrusion width (EW)

	Setting	Value
	Perimeter	2
**Cortex**	Infill pattern	linear
	Infill density	100%
	EW Outer contour	0.6 mm
	EW contour	0.6 mm
	EW Infill	0.45 mm
	EW Massive infill	0.6 mm
**Spongiosa**	Infill pattern	gyroid
	Infill density	7/12/16/27%
	Perimeter	0

### Mechanical testing

**Fig. 6:**
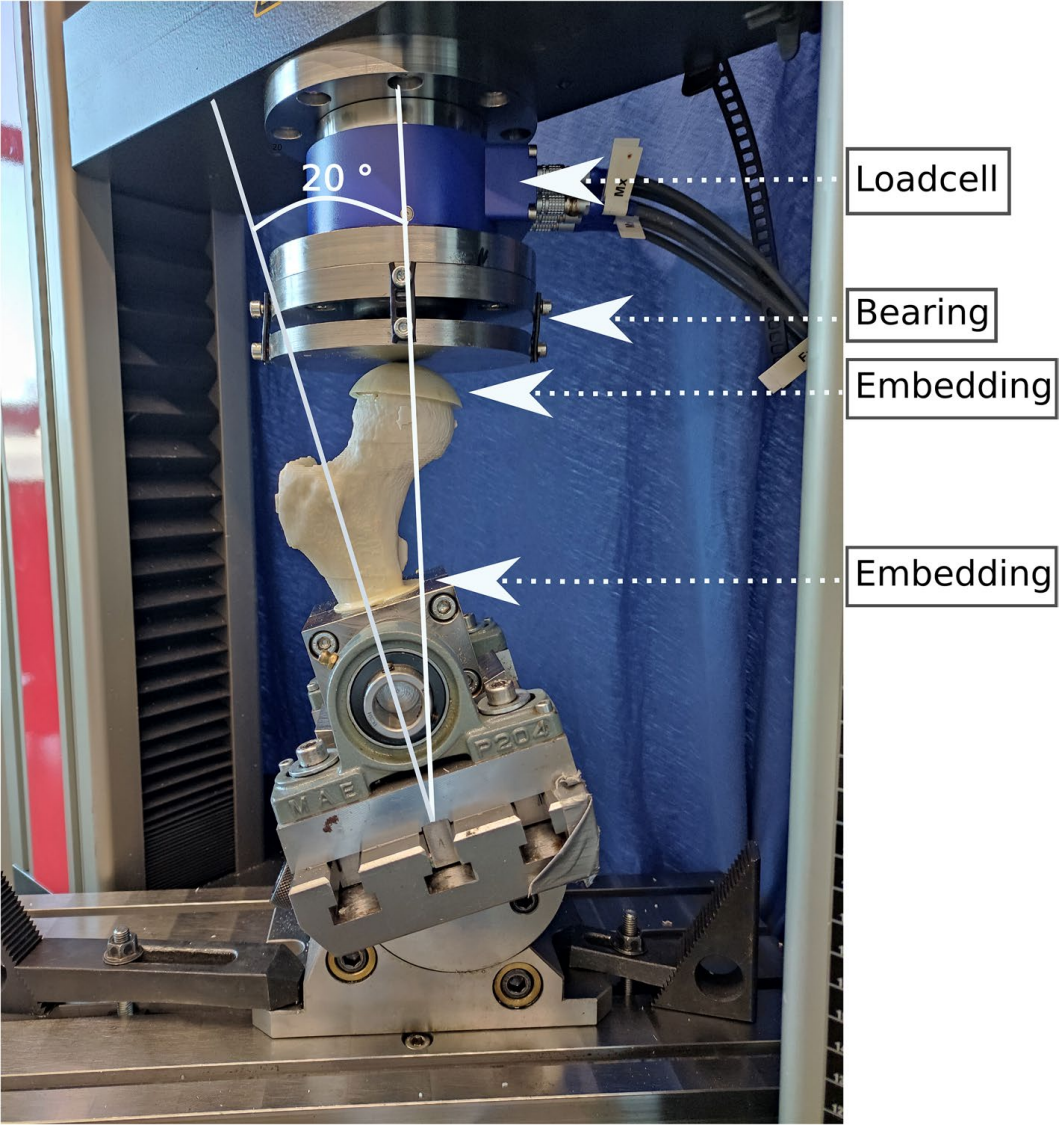
Biomechanical Test Set-Up. 3D printed bone embedded in polyurethane foam declined for 20° to simulate stance position during a gait cycle

The bones were tested at a 20° angle between loading direction and proximal shaft axis as shown in Fig. 6, simulating one-legged stance phase in a gait cycle according to Dall’Ara et al. [33]. This angle was chosen due to the human bones tested by Dall’Ara et al. [33] at 20° and studies simulating one-legged stance phase often given an angle between 7° and 25° to femurs involved in biomechanical testing [31, 40–44]. The compression tests were performed on a Z030 machine (ZwickRoell GmbH & Co. KG, D-89079 Ulm, Germany) with a compression rate of 5 mm/min until failure and position were captured at 100 Hz while recording machine displacement *u* and the force *F*.

**Fig. 7:**
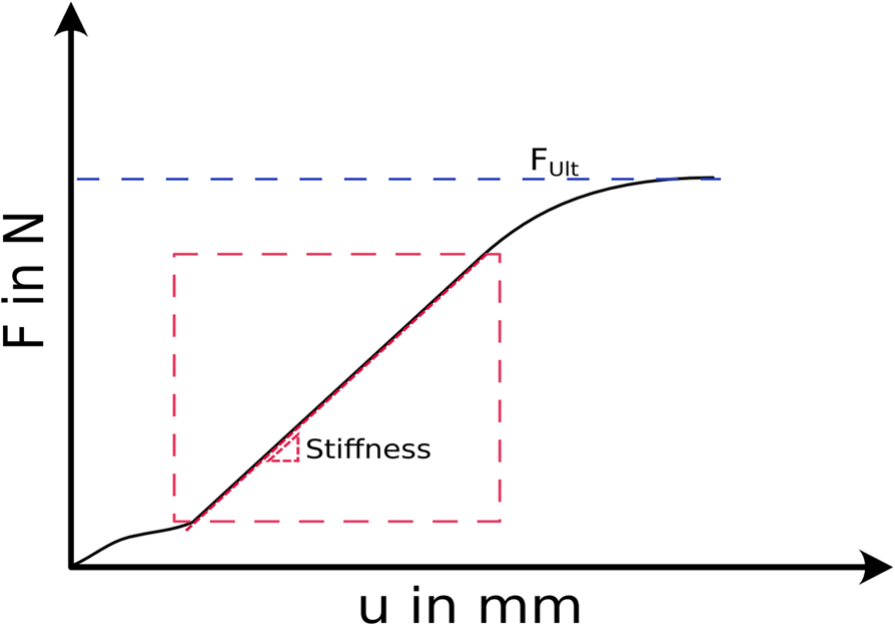
Schematic force-displacement curve with marked ultimate force (blue), and linear region for the best linear fit (red)

The femoral ultimate force *F*_*Ult*_ was defined as the maximum compressive load as shown on basis of the blue dashed line in Fig. 7. The spring stiffness *k* was evaluated by taking the best linear fit determined by the maximum R^2^ value of the “force-displacement” curve, indicated by the schematic red area marked in Fig. 7.

## Results

**Fig. 8:**
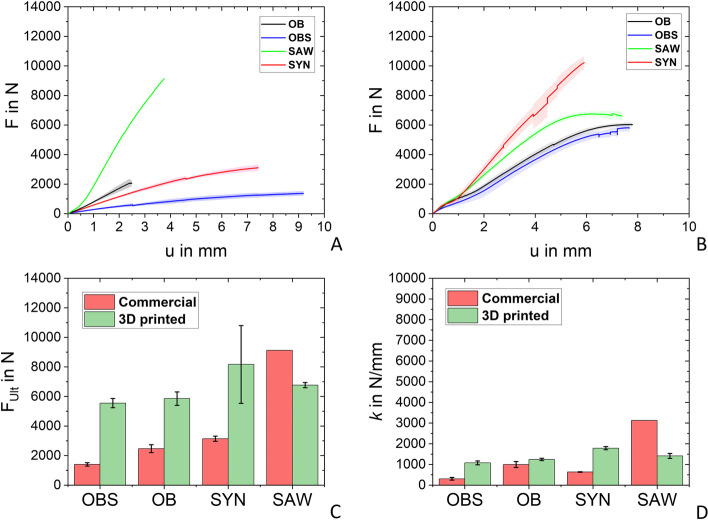
**A** mean force (line) plotted over the displacement and its standard deviation (shaded region) for commercial artificial bones. **B** mean force (line) plotted over the displacement of the 3D printed bones based on generic bone geometry and its standard deviation (shaded region). **C** ultimate forces **D** average spring stiffness

The resulting average force-displacement curves and standard deviation resulting from the compression tests were plotted for the commercial bones (Fig. 8A) and the 3D printed bones of the commercial bone analogs (Fig. 8B) as well as the human bone analogs (Fig. 10B). Whereas for the commercial bones two samples in the case of OB, OBS, and SYN were tested and one composite bone (SAW).

### Commercial bones

The experimental curves of the commercial bones (Fig. 8A) exhibit displacements *u* between 2 mm and 9 mm before failure occurred and the range of ultimate force *F*_Ult_ from 1404 N (OBS) to 9119 N (SAW) as shown in Fig. 8C and Table 4. The spring stiffness *k* of the composite SAW bones is high (3127 N/mm) in comparison to the PU-based bones (OB: 997 N/mm, OBS: 307 N/mm, SYN: 635 N/mm) as listed in Table 4 and shown in Fig. 8D.

**Table 4:** Results of the compression tests for artificial commercial bones (*n* = 2) and their 3D printed counterpart (*n* = 6)

		Commercial		3D printed	
		***F***_**Ult**_ in N	*k* in N/mm	***F***_**Ult**_ in N	*k* in N/mm
**Sample**	**OB**	2470.3 ± 268.5	996.6 ± 145.5	5854.2 ± 458.7	1243.3 ± 54.9
	**OBS**	1403.2 ± 112.4	306.8 ± 65.6	5551.6 ± 313.3	1077 ± 94.6
	**SYN**	3144.3 ± 174.3	635.3 ± 12.8	8166.2 ± 2635.0	1787 ± 76.9
	**SAW**	9119.3	3127.5	6766.0 ± 175.0	1412.3 ± 118

The printed bones exhibit a non-linear region at the beginning of the compression test as shown in Fig. 8B due to adapting the contact area with the force plate. Therefore, the linear region after the toe region was used for spring stiffness evaluations. In general, the printed bones fracture all between 6- 8 mm of displacement as shown in Fig. 8B. Also, the deviation between experimental *F*_Ult_ is less (Fig. 8C), with values ranging from the lowest 5552 N (OBS) to 8166 N (SYN). All 3D printed bones based on the generic geometry show an average spring stiffness between 1077 N/mm (OBS) and 1243 N/mm (SAW) and are listed in Table 4 as well as demonstrated in Fig. 8D.

**Fig. 9:**
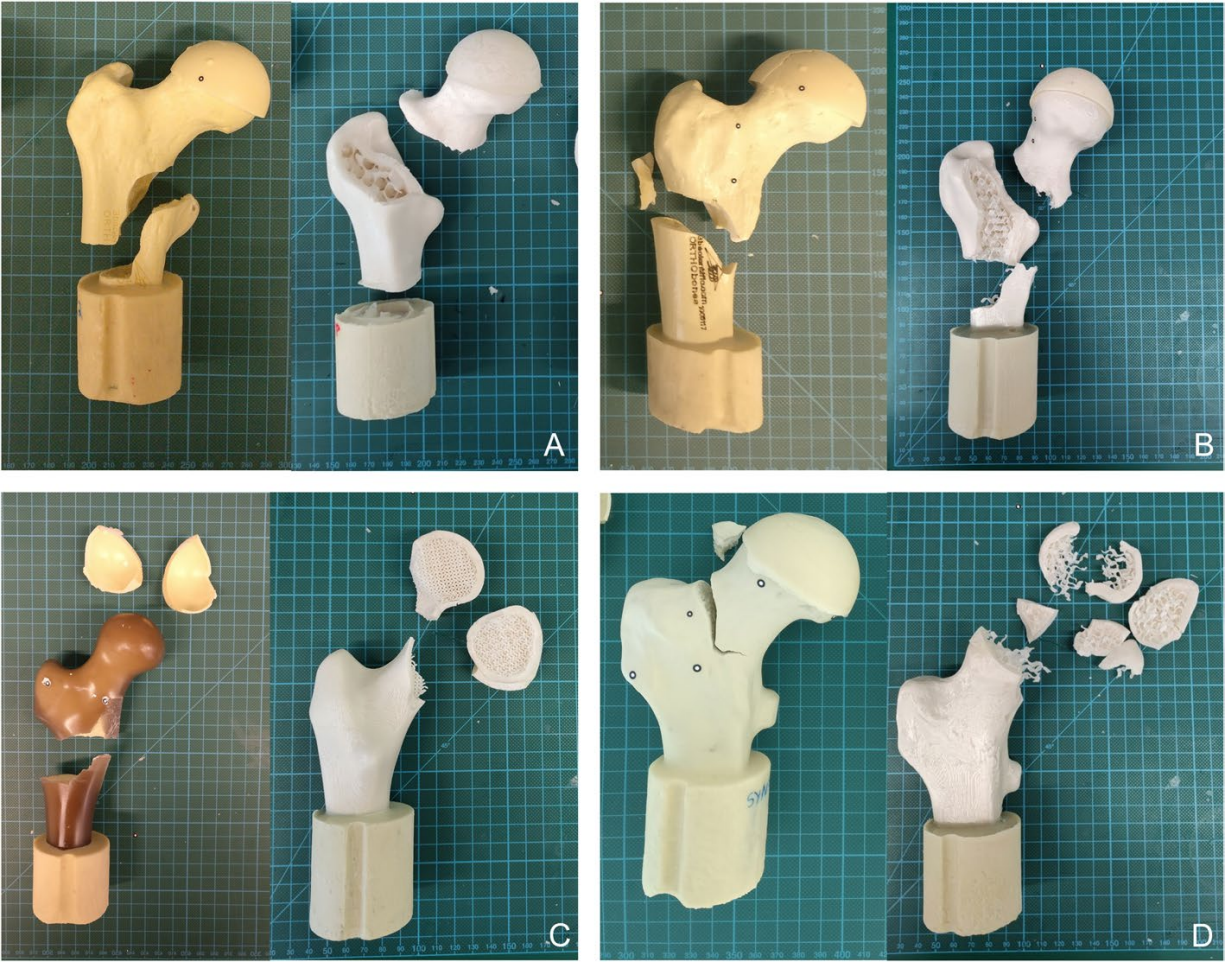
Fracture Pattern of the commercial bones (left) in comparison to the 3D printed bones (right). **A** OB **B** OBS **C** SAW **D** SYN

Almost all 3D printed bones showed a fracture either in the femoral neck region, as shown in Fig. 9C and Fig. 9D, or the trochanteric region (Fig. 9A and Fig. 9B). Whereas the commercial counterpart mainly broke in the femoral shaft region (Fig. 9). Except for the SYN sample, which also fractured in the femoral neck region. The fracture pattern of the OB, OBS and SAW commercial sample can be classified as a simple fracture in the proximal diaphyseal segment of the femur (32A) according to the AO/OTA fracture and dislocation classifications [45]. While the fractures in the femoral neck region of the 3D printed counterparts classify as transcervical femoral neck fractures (31B2) in the case of SAW (Fig. 9C right) and SYN (Fig. 9D right). Concerning the fracture pattern of the OB (Fig. 9A) and OBS (Fig. 9B), 3D printed bones, can be classified as 31A fractures [45]. According to literature, the most common fracture sites of the elderly are either intertrochanteric fractures [46, 47] or fractures located at the femoral neck [47], which are successfully simulated in this study.

### Human bones

**Fig. 10:**
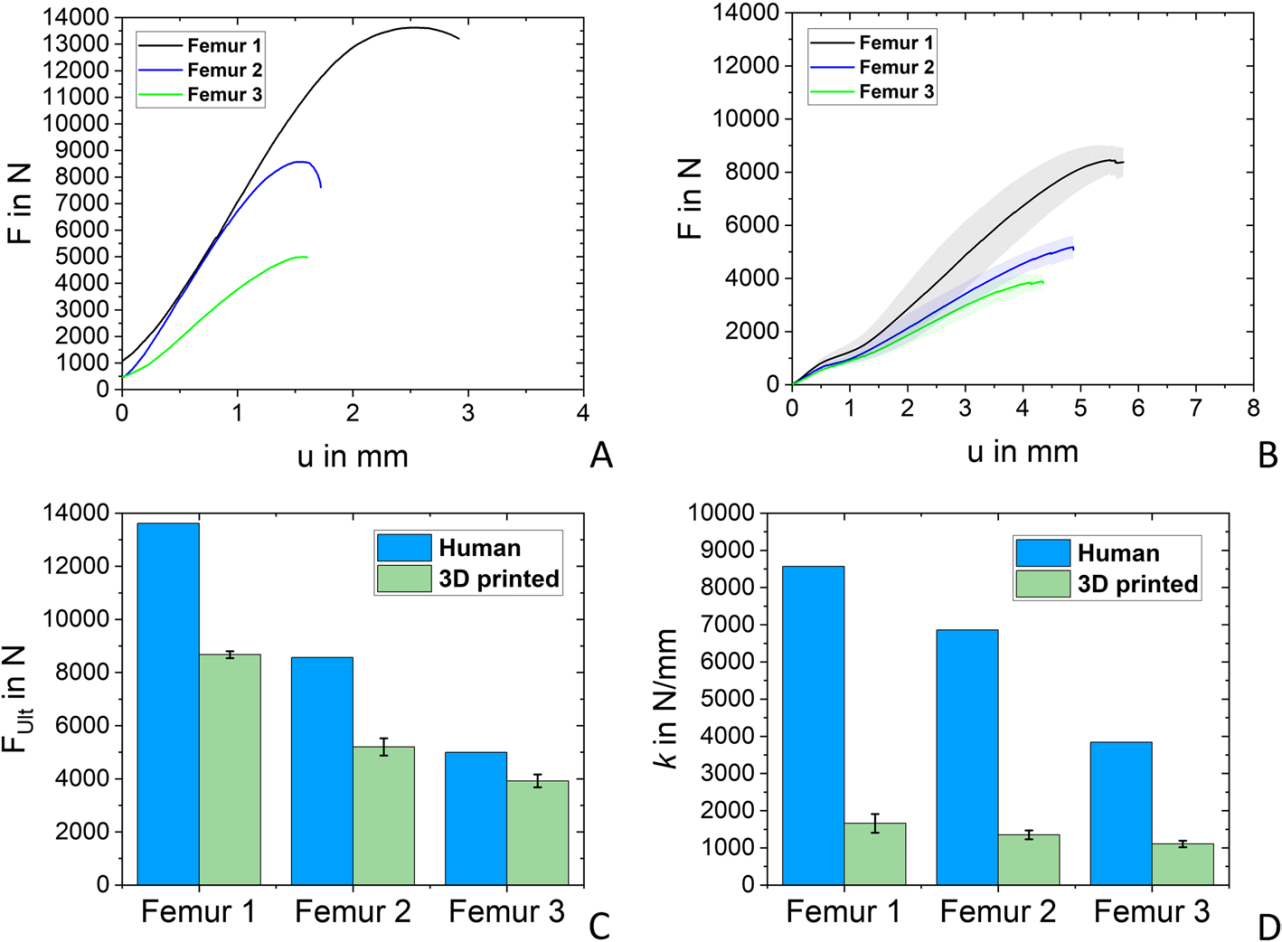
**A **experimental curves of the human bones [33] **B** mean force plotted (line) over the displacement of the 3D printed bones based on human bone geometry and its standard deviation (shaded region)*.* **C** reached ultimate forces of the 3D printed bones and standard deviation in comparison to human bones [33]*.* **D** average spring stiffness of 3D printed human femur analogs in comparison to human bones [33]

**Fig. 11:**
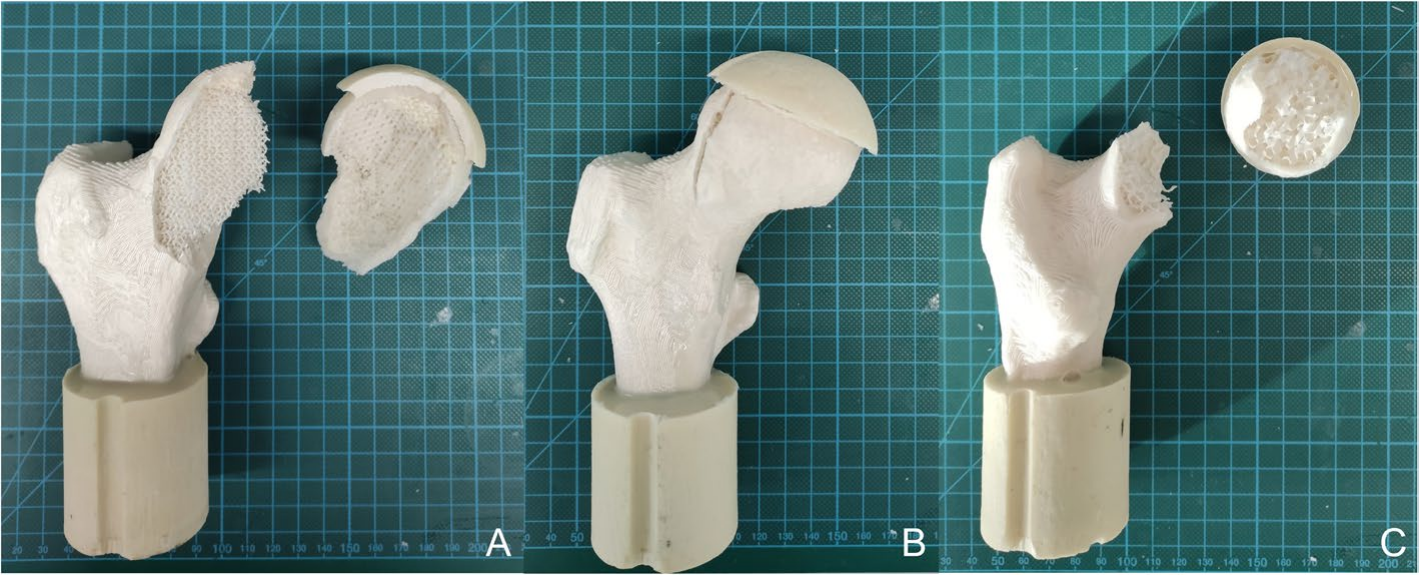
Fracture Pattern of 3D printed femoral bone based on human femoral bone geometry **A** Femur 1 **B** Femur 2 **C** Femur 3

The force-displacement curves of the real human bones from Dall’ Ara *et. al*. [33] are shown in Fig. 10A. The experimental force-displacement curve of the 3D printed bones based on the geometry of human femoral bones is visualized in Fig. 10B. Fracture of the samples occurred after 4.5 to 6 mm displacement (Fig. 10B) while withstanding *F*_Ult_ of 3921 N (Femur 3), 5197 N (Femur 2) up to 8677 N of Femur 1 as shown in Fig. 10C. Values of the real human femoral bones were taken from a previous study [33] and are included in the diagrams of Figs. 10C and 10D as well as listed in Table 5. Also visible in the graph of Fig. 10B is a non-linear region at very low loads. For further calculations of the spring stiffness of the samples, the linear region after this region was analyzed resulting in spring stiffnesses listed in Table 5 of 1104 N/mm (Femur3), 1352 N/mm (Femur 2), and 1600 N/mm for Femur 1. Furthermore, the fracture pattern of the 3D printed bones was evaluated and resulted in a fracture in the femoral head down to the femoral neck region, classified as 31C [45] in the case of Femur 1 (Fig. 11A) and a fracture in the femoral neck (31B) in case of Femur 3 (Fig. 11C). Whereas Femur 2 showed a fracture through the femoral head down to the neck (Fig. 11B), due to layer splitting.

**Table 5:** Results of the compression tests for human bones [33] (*n* = 1) and their 3D printed counterpart (*n* = 6)

		Human [33]		3D printed	
		***F***_**Ult**_ in N	*k* in N/mm	***F***_**Ult**_ in N	*k* in N/mm
**Sample**	**Femur 1**	13,620	8568	8676.5 ± 130.5	1659.5 ± 251.3
	**Femur 2**	8568	6861	5197.2 ± 324.1	1351.5 ± 119.3
	**Femur 3**	4992	3837	3920.9 ± 243.6	1104.4 ± 89.8

## Discussion

The present study aimed to investigate the possibility of using 3D printing for the fabrication of femoral bone surrogates and compare their mechanical behavior in terms of ultimate force *F*_Ult_ and spring stiffness *k* with artificial commercially available femoral bones. Additionally, the capability of printing patient-specific geometries was investigated. A comparison of the mechanical behavior of donor bones [33] with their 3D printed analogs was performed.

The bones were printed with a commercial “low budget” FMD 3D printer and took an average of 14 h to print for 3.5€. In general, 3D printed bones showed realistic fracture patterns (Figs. 9 and 11), better strengths than inexpensive commercial bones, and similar behavior to expensive SAW bones. Compared to real human bones, the obtained strength of 3D printed bones is quite acceptable, but the stiffnesses are 3–4 times too low.

### Commercial vs. 3D printed bones

Regarding the ultimate force, Fig. 8B shows little fluctuations within the curves of the 3D printed bones. These fluctuations arise from layer splitting which can occur using FDM.

When looking at the polyurethane-based bones (OB, OBS, and SYN) withstand only one-half of the force compared to their 3D printed counterparts. As expected, the cheaper and lower quality commercial bone OBS endured lower forces (1000 N) compared to its geometrical equal bone OB (2470 N). In comparison to this observation, the different infill densities of the printed samples of OBS and OB do not influence the outcome as much as the different foam used. When comparing the commercial OB and SYN samples, which have similar mechanically performing foams as spongiosa compensation, the SYN sample withstands approximately 700 N more than the OB samples. Also, the comparison between the 3D printed OB and SYN samples shows a deviation in withstanding different ultimate forces, despite sharing the same infill density. This observation, concerning the commercial as well as the 3D printed bones, might be a result of the different geometries of the bones since the SYN bones have a thicker femoral neck. Comparing the commercial polyurethane-based bones with artificial bones reported in literature, the range of ultimate force (1403–3144 N) is considerably beneath the range of 5528–11,109 N [44] and the average ultimate force of 7590 [48]. Only the SAW bone is comparable with these results by exhibiting an ultimate force of 9119 N. Whereas, the 3D printed commercial replicated bones could keep up with previous tested artificial bones by withstanding forces between 5552 N (OBS) and 8166 N (SYN).

In this study, also a look at the spring stiffness of the commercial as well as the 3D printed bones was taken. The influence of the foam representing the artificial spongiosa on the spring stiffness is clearly shown when comparing the OB and the geometrical identical cheaper bone OBS. This influence vanishes when looking at the comparison between the 3D printed counterparts of these bones. Indicating that the infill density does not influence the spring stiffness as much as the geometry concerning the 3D printed bones. This finding is supported by the results for the SAW sample, with an infill density of 27% (in comparison to 7–12%), which does not increase the spring stiffness significantly. The composite commercial SAW bone exhibits the spring stiffness range of the polyurethane-based bones clearly and is also stiffer than every 3D printed bone tested in this study. It is worth mentioning that the cortical thickness influences the spring stiffness of the bone [49]. In this study, only the spongiosa were modeled in terms of mechanical properties. The mechanical characteristics of the commercial cortex were not taken into account, which could be an explanation for the lower spring stiffness of the 3D printed SAW bone in comparison to the commercial composite bone. Nevertheless, tested bone in the present study exceeds stiffnesses considerably compared to previous studies, reporting spring stiffnesses between 1290 - 2530 N/mm [23, 31, 44, 48, 50]. The deviation of spring stiffness compared to literature can be traced back to different sample preparation, including the embedding process and the chosen angle of load application.

### Human vs. 3D printed bones

The patient-specific modeled 3D printed bones all endure less load (Femur 3: 3920 N, Femur 2: 5197 N, and Femur 1: 8677 N) compared to the real human bones (Fig. 10B and Table 5), which was to be expected due to the bone being a highly complex composite material in comparison to PLA. However, the samples tested in this study lie well in the range of reported literature values for human bones which range from an average of 3500 N (for 20 samples tested) [51] to 6600 N (with a broad scatter range 3780–12,396 N) [52]. In general, a significant variation in mechanical properties of human bones is observed as values of up to 9196 ± 3177 N [40] and 8890 ± 377 N [43] are also reported. A possible explanation for this is a variation in testing setup as different representations of the stance phase (consequentially testing angles) are used by each research group. Furthermore, a dependence on the gender of the donor was found by Link *et. al.*, who showed that male femoral bones were typically stronger by 40% [43]. Nevertheless, it was possible to recreate the trend observed for the ultimate force in the human donor bones by the 3D-printed analogs.

Concerning stiffnesses reached by the 3D printed bones based on real human femoral geometries range from 1104 up to 1660 N/mm (Table 5). Compared to the actual bones tested by Dall’Ara *et. al*. [33] (Table 5), the 3D printed Femur 1 and Femur 2 samples only reach a fifth of the real bone stiffnesses, whereas Femur 3 accomplished a third of the stiffness of the donor’s bone. An explanation for this is, bone is a highly complex composite material with an approximated Young’s modulus of the cortex of 17.4 GPa [53] in comparison to the used PLA with an Young’s modulus of 3.5 GPa [54], which is almost 5 times lower.

Nevertheless, different stiffnesses of human bones are found in literature [23, 51]. Researcher also tested femurs at 20° declined to represent stance phase with a resulting stiffness of 1280 N/mm [51], this value is indeed comparable with the spring stiffnesses found in the present study. Also, elderly human femoral bones were tested resulting in an average stiffness of only 757 N/mm [23], which is around 400 N/mm less stiff than 3D printed human femoral bones in the present study. The lower stiffness reported could be a result of the whole bone test setup, which makes the bone “softer” in comparison to the truncated femoral test setup [23].

Besides the pleasant results of 3D printed bones in comparison to commercial and human bones, a few limitations must be mentioned. The results are limited to intact proximal human femurs, which are tested under the physiological condition representing the stance phase during human gait. Furthermore, this study was limited to the use of a low-budget FDM printer with standard PLA as a filament. Changes in the whole process (printer maintenance like nozzle changes, storage and age of filament rolls, lab temperatures and humidity) could result in higher, at the moment not explainable, standard deviations as shown in Fig. 8C concerning the SYN samples. Therefore, the chosen printer, the printing settings as well as the chosen printing direction could influence the obtained results considerably.

## Conclusions

Nevertheless, this study confirmed that 3D printing is a promising tool to produce femoral bone surrogates. The standard deviation within a tested sample group is quite low, which indicates consistency throughout the printing procedure as well as the mechanical testing.

Especially in comparison with existing polyurethane-based surrogates, the 3D printed bones perform mechanically better regarding ultimate forces (~ 5550–8200 N) and stiffnesses (~ 1100–1400 N/mm). Only the composite bone of generic geometry mechanically performed better than its 3D printed counterpart.

In comparison to real human bones, the printed ones showed the same trend of increasing ultimate forces with increasing bone density. But more (stiffness) or less (strength) off compared to the response of real human bones. New filaments are necessary with a slightly higher strength, but considerable higher elasticity is required.

In conclusion, if the geometries and a 3D printer are available, bones can be printed overnight, and the only cost of the material incur, and a multiplicity of bones can be produced inexpensively. Patient-specific studies will be possible, and many other possibilities will open in the future.

## Data Availability

The datasets used and analyzed during the current study are available from the corresponding author on reasonable request.

## Abbreviations

AM: Additive Manufacturing
BV: Bone Volume
EW: Extrusion Width
FDM: Fused Deposition Modeling
*F*_*Ult*_: Ultimate Force
μ-CT: Micro-computed Tomography
OB: Orthobone
OBS: Orthobone_Standard
PLA: Polylactide
PU: Polyurethane
q-CT: Quantitative Computed Tomography
SAW: Sawbone
SLA: Stereolithography
SLS: Selective Laser Sintering
STL: Standard triangulated surfaces
SYN: SYNBONE
TV: Total bone volume
